# Third-Body Dynamics and Tribo-Oxidation Mechanism in Fretting Degradation of an Inconel 718/304 Stainless Steel O-Ring Seal

**DOI:** 10.3390/ma19194137

**Published:** 2026-09-28

**Authors:** Bo Yang, Chaojun Deng, Linyuan Kuang, Zeyuan Yu, Ying Luo

**Affiliations:** 1 School of Mechanical Engineering, Sichuan University, Chengdu 610065, China; 2 National Key Laboratory of Nuclear Reactor Technology, Nuclear Power Institute of China, Chengdu 610041, China

**Keywords:** nuclear engineering, reactor pressure vessel, metal O-ring seal, static seal, fretting wear test, fractal analysis

## Abstract

Fretting wear at metal O-ring sealing interfaces involves the coupled processes of debris generation, entrapment, and oxidation. Existing accelerated life testing relies on similarity theory, yet it overlooks the dominant role of third-body behavior in governing wear regime transitions, so equivalence between test and service conditions lacks a physical basis. This study investigates reciprocating fretting wear of an Inconel 718/304 stainless steel pair and reinterprets wear evolution using third-body dynamics. Laser scanning confocal microscopy, energy-dispersive spectroscopy, and real-time friction monitoring reveal three distinct stages: debris generation with mild damage, oxide layer formation with steady wear, and oxide layer fracture with material spalling. The friction coefficient passes through running-in, steady-state, and sharp-rise phases, while oxygen content on the wear track rises from 2.1 wt.% to 18.3 wt.%. One-way ANOVA shows significant differences among all stages. Based on Berthier’s theory, a state-evolution model is developed that treats third-body oxidation degree and cumulative friction energy dissipation as equivalence criteria. Friction energy and wear mass correlate linearly (R^2^ = 0.94), giving an effective wear coefficient of 6.3 × 10^−7^ mg/J and acceleration exponents of m_1_ = 9.77 for pressure and m_2_ = −8.71 for frequency. Independent validation shows that the model compresses test duration by 51%, with relative errors below 18% for wear mass, fractal dimension, and fractal roughness. By shifting the focus of accelerated testing from dimensional analysis to mechanism-preserving state tracking, this work provides a failure analysis framework that identifies the root cause of seal degradation as third-body oxidative spalling and offers practical preventive actions for nuclear metal seal reliability assessment.

## 1. Introduction

### 1.1. Background and Motivation

Several operational experience databases and fault diagnosis studies document metal O-ring seal failures in nuclear reactors [1]. Researchers have systematically classified leakage fault modes of reactor coolant pumps, and seal degradation is a primary contributor to coolant boundary integrity loss. Thermal shock and uneven deformation during transient operations reduce contact pressure on sealing surfaces, which leads to leakage escalation in marine and land-based nuclear systems [2]. IAEA aging management guidelines document long-term aging degradation of metallic and organic sealing components under operational exposure to elevated temperatures, radiation, and environmental stress [3]. Post-service examinations of failed nuclear components also reveal material degradation mechanisms such as nickel leaching from pump seal wear rings under reactor coolant conditions [4]. In these events, leakage escalation traces to sealing surface degradation rather than bulk material rupture, which shows that surface-state evolution is the dominant failure mode. Despite this, failure analysis of metal seals has traditionally focused on post-leakage macroscopic inspection or data-driven fault diagnosis [4], with limited attention to the progressive micro-scale damage accumulation that precedes functional failure. This study tackles this problem by treating fretting wear as a failure precursor and investigating the underlying mechanism transitions through third-body dynamics.

We focus on nickel-based alloys and stainless steels. This material pair is the most widely used in reactor seals. Research on this pair has grown over the past three years. Several groups have studied how fretting damage of Inconel 718 evolves with temperature [5]. They found the protective and subsequently detrimental roles of tribo-oxide layers [6,7]. They also quantified how manufacturing route alters fretting response [8,9,10,11,12]. The routes include wrought, laser cladding, selective laser melting, and directed energy deposition. For the Inconel 690/304 stainless steel pair used in pressurized water reactors, fretting destroys the native passive film. It triggers a sudden loss of corrosion resistance. This shows that surface-state evolution, not bulk wear volume, controls functional failure [13]. Related work on nickel-based superalloy tribology has established that compaction and sintering of oxidized debris into a glaze layer can reduce wear rates by an order of magnitude at elevated temperatures [14,15]. Spinel-type oxides can even provide sustained high-temperature self-lubrication [16]. A recent review pulls these findings together. In this view, debris generation, retention, oxidation, and re-attachment at the interface determine the wear mechanism and rate [17].

The design life of a metal O-ring is often several decades. Full-duration testing is impractical because of cost and time. Accelerated testing, if properly grounded in failure mechanism preservation, provides a practical approach to predicting seal degradation within design timeframes. It compresses test duration by elevating load, frequency, or environmental severity, and the results are extrapolated back to service conditions. For metal O-ring seals, an accelerated fretting wear test protocol for the Inconel 718/304 stainless steel pair has only recently been proposed and validated. It remains rooted in classical similarity theory. Equivalence is judged by matching dimensionless groups and reproducing equal wear amounts. Whether such equivalence preserves the actual damage mechanism is still unclear.

### 1.2. Limitations of Existing Work

Research on fretting wear of metal seals and their candidate materials falls into two broad categories: phenomenological studies and model-based approaches. The first is phenomenological. Studies use scanning electron microscopy, energy-dispersive spectroscopy, and white-light interferometry to document wear morphology and debris composition. They then classify wear mechanisms into adhesive, abrasive, and oxidative regimes [5,6,7,8,9,10,11,12]. These studies provide detailed observations but lack predictive capability. The mechanism maps they produce apply only to the specific test conditions. They cannot be transferred to accelerated conditions.

The second track is model-based. Finite element and semi-analytical models predict contact pressure, slip amplitude, and frictional energy dissipation [18]. Archard-type or energy-based wear laws are fitted to measured wear volumes. Recent high-temperature studies show that the wear coefficient changes with test conditions. It changes by a factor of 10 to 100 as the interfacial oxide layer forms, compacts, and fractures [14,16]. Wear depends on the state of the third body. This is the layer of debris, oxides, and transfer films trapped between the two first bodies. Berthier introduced this concept. Modern characterization has shown it [17,19].

Berthier’s third-body theory describes the contact through three competing flows. Source flow is debris detachment from the first bodies. Internal flow is crushing, mixing, and oxidation of debris within the contact. Ejection flow is debris escape from the contact edges [19]. Under fretting conditions, small displacement amplitudes restrict debris ejection. A third-body layer forms inside the contact. Its composition, thickness, and rheology evolve with time. They determine whether the interface experiences mild adhesive wear, protective glaze-layer sliding, or severe oxidative spalling [14,17,18]. Third-body dynamics has rarely been incorporated into accelerated testing methodology, despite its proven explanatory power. Existing ALT practice for seals and wear components still relies on similarity criteria derived from geometry, load, and motion [4]. It implicitly assumes that the wear mechanism is invariant under acceleration. The recent literature contradicts this assumption. Increasing frequency alters debris residence time and oxidation degree [6,7]; increasing load raises the debris generation rate and layer thickness [8,14]; and increasing temperature accelerates oxidation kinetics by orders of magnitude [5,16]. Two tests may yield identical wear volumes yet exhibit entirely different third-body states and remaining sealing lives.

### 1.3. Core Idea and Contributions

We advance the following premise. Equivalence in accelerated fretting wear should be based on third-body dynamics. It should not rest merely on geometric and kinematic similarity. Two operating conditions are equivalent if they produce a third-body layer of the same oxidation degree, same thickness, and same fractal character of the worn surface. They must also produce the same cumulative friction energy dissipation per unit area. This premise shifts the focus of accelerated testing from dimensional analysis to state tracking. The state variables are measurable. Oxygen content is measurable by EDS, layer morphology by confocal microscopy, and friction energy from real-time tangential force records. Their equivalence can be verified independently of wear mass.

Based on this premise (Figure 1), we pursue four failure analysis tasks. To test this idea, we first ran systematic fretting wear experiments on an Inconel 718/304 stainless steel pair, combining surface morphology, mass loss, debris chemistry, and real-time friction monitoring to track third-body evolution. We then built a wear-state model based on Berthier’s theory, incorporating debris residence time, parabolic oxidation kinetics, and cumulative friction energy dissipation. From this model, we derived new equivalence criteria for accelerated testing—criteria that depend on third-body oxidation degree and cumulative friction energy rather than on geometric similarity alone. Finally, we checked the model against an independent validation test to see whether shortened test durations preserve damage-state equivalence in wear mass, surface fractal dimension, and fractal roughness.

This figure was created with the assistance of KIMI, a generative AI tool; the authors reviewed and edited the output and take full responsibility for the content.

## 2. Materials and Methods

### 2.1. Materials and Specimens

Inconel 718 alloy and 304 stainless steel were chosen to represent the sealing pair of a reactor pressure-vessel O-ring. The upper specimen was Inconel 718, Φ6 × 7 mm. The lower specimen was 304 stainless steel, Φ19 × 8 mm. The Inconel 718 specimens were machined from Special Metals INCONEL^®^ 718 bar [20], solution-treated and aged per AMS 5662 [21]/ASTM B637 [22]. The 304 stainless steel specimens were supplied as solution-annealed plates per ASTM A276/A240 [23]. No additional heat treatment was applied. Mechanical properties and chemical composition are listed in Table 1 [24] and Table 2 [24].

### 2.2. Fretting Wear Tests

We ran tests on an Rtec MFT-5000 reciprocating fretting tribometer (Rtec Instruments, Inc., San Jose, CA, USA). The upper specimen remained fixed; the lower specimen reciprocated horizontally. All tests ran in ambient air (20–25 °C) under dry conditions. Friction force and displacement were recorded at 10 kHz.

Four test conditions were designed for calibration and mechanism tracking (Table 3), plus one independent validation condition (Condition 4) (Table 3). Condition 1 served as the baseline, with intermediate sampling at 0, 4, 8, and 12 h to track full wear evolution. Conditions 2, 3, and 5 provided data for model calibration. Condition 4 served as an independent validation.

### 2.3. Characterization Methods

Surface morphology. We captured three-dimensional surface maps with an OLYMPUS 5000 laser scanning confocal microscope. The fractal parameters were extracted by the structure function method [27]: for a self-affine surface, the structure function S(τ) = 〈[z(x + τ) − z(x)]^2^〉 scales with the lag distance τ as S(τ) ∝ τ^(4 − 2D), so that the fractal dimension D (2 < D < 3 for a surface) is obtained from the slope of the log S(τ)–log τ plot, and the fractal roughness parameter G is obtained from its intercept, characterizing the amplitude of the self-affine roughness at the unit length scale. D describes the complexity of the surface (how completely the structure fills the space across scales), whereas G describes the magnitude of the multi-scale height fluctuations; the two parameters are complementary and together provide a scale-independent characterization of worn-surface topography [27,28]. For statistical robustness, five non-overlapping fields of view (each 640 × 480 μm^2^) were scanned per specimen at each sampling point. We extracted ISO 25178 [29] areal parameters using MountainsMap software(Digital Surf, Besançon, France, version 10.1) after form removal (F-operator) and Gaussian S-filter ($\lambda_{c}\;=\;80$ μm). The values of D and G are scale-dependent [30]; the reported parameters are tied to the present configuration (640 × 480 μm^2^ field of view, λ_c_ = 80 μm) and should be re-evaluated when transferring the criteria to other measurement systems.

Mass loss. A METTLER TOLEDO (Greifensee, Switzerland) analytical balance (resolution 0.01 mg) measured specimen mass before and after testing. We computed the wear amount $\delta_{t}$ from mass loss. We tested each condition with three independent replicates (n = 3).

Chemical composition. EDS area scans on the 0, 4, 8, and 12 h specimens from Condition 1 quantified O, Fe, Cr, and Ni distributions inside the wear track. The oxygen content increment ΔO served as a proxy for third-body oxidation degree. We analyzed five distinct regions per specimen (each 100 × 80 μm^2^) to account for spatial heterogeneity.

Friction coefficient. We averaged real-time friction–displacement curves per cycle to track friction evolution over time. The reported steady-state friction coefficient represents the mean of the last 30 min of each stage, averaged across three replicate tests.

### 2.4. Statistical Analysis

All quantitative measurements included at least three independent replicates, and we report results as mean ± standard deviation (SD). We performed one-way analysis of variance (ANOVA) to assess differences among wear stages (0, 4, 8, and 12 h), followed by Tukey’s honestly significant difference (HSD) post hoc test for pairwise comparisons. Normality was verified using the Shapiro–Wilk test, and homogeneity of variance was checked using Levene’s test. Statistical significance was set at *p* < 0.05. We quantified effect size using eta-squared (η^2^). All analyses used Python 3.9 with SciPy and statsmodels libraries. The choice of one-way ANOVA with Tukey HSD post hoc testing follows from the structure of the data: the response variables (oxygen content, fractal dimension, fractal roughness) are continuous measurements on specimens grouped by a single categorical factor (wear time), and the assumptions of normality (Shapiro–Wilk) and homogeneity of variance (Levene) were verified for all comparisons. For such balanced, single-factor designs with continuous responses, classical parametric statistics remain the standard and most interpretable approach. We note, however, that complementary multi-criteria decision-making and machine-learning methods—including TOPSIS, response surface methodology, decision trees, support vector machines, and random forests—are increasingly used in tribological studies for multi-objective evaluation, condition ranking, and prediction when designs are non-orthogonal, or responses are categorical [31,32,33]. These approaches may be incorporated in future work to optimize test-condition selection and to classify degradation states across a wider parameter space.

For EDS elemental analysis and surface-topography characterization, we employed a nested sampling design: three independent specimens per wear stage, each providing five spatially distinct measurement regions (fields). This yields n = 15 observations per group. Because regions within the same specimen are technically correlated, we first assessed the intraclass correlation coefficient (ICC) via a random-intercept linear mixed-effects model (LMM) with specimen as a random effect. The ICC was <0.12 for oxygen content and <0.08 for fractal roughness, showing that >88% of the total variance arises between wear stages rather than within specimens. Consequently, one-way ANOVA at the region level (denominator df = 56) is reported as a robust approximation, and the LMM corroborated all key conclusions (Type III Wald χ^2^ test). We performed post hoc pairwise comparisons using Tukey HSD on the marginal least-squares means.

## 3. Theory Calculation

### 3.1. The Role of Third Bodies in Fretting Wear

In fretting wear, the third body comprises wear debris, oxides, and transfer films that detach from the two base materials and participate in subsequent friction processes inside the contact zone. Berthier [19] describes its behavior through three competing flows:

Source flow. Base material fractures and detaches under shear stress, producing fresh debris. The generation rate depends on contact stress, material hardness, and interfacial adhesion.

Internal flow. Debris is repeatedly crushed and mixed inside the contact, forming a third-body layer with its own thickness and mechanical properties. Residence time depends on displacement amplitude, frequency, and contact geometry. Small fretting displacements limit debris ejection. Residence times are much longer than those in sliding wear.

Ejection flow. When the third-body layer exceeds a critical thickness or experiences sufficient shear, debris escapes from the contact edge. Ejection rate depends on displacement amplitude, frequency, and layer cohesion.

The balance of these three flows determines the third-body state and, in turn, the wear mechanism: When generation outpaces retention, debris escapes quickly. Wear remains mild and adhesive or abrasive. When residence time is long enough, debris oxidizes and compacts into a protective glaze layer. Wear rate drops. When the oxide layer grows too thick or too brittle, it fractures under shear. Hard oxide particles form, and severe abrasive wear sets in.

### 3.2. Oxidation Kinetics of the Third Body

Metal debris oxidizes in air following parabolic kinetics. For 304 stainless steel, the main oxides are Fe_2_O_3_, Fe_3_O_4_, and Cr_2_O_3_. Let θ (0 ≤ θ ≤ 1) denote the average oxidation degree of the third-body layer. Its evolution rate is:

(1) $$\frac{d\theta }{dt}\;=\;k_{ox}\cdot \left(1-\theta \right)\cdot \exp\left(-\frac{E_{a}}{RT_{eff}}\right)\cdot f_{renew}$$

Here, $k_{ox}$ is the oxidation rate constant, $E_{a}$ the activation energy, $T_{eff}$ the effective interface temperature raised by friction, and $f_{renew}$ the surface renewal frequency, proportional to the reciprocating frequency. Equation (1) adopts the classical saturation-type (parabolic-limit) oxidation kinetics, in which the oxidation rate is proportional to the remaining unoxidized fraction (1−θ), following the Wagner theory of metal oxidation [34]; the exponential temperature dependence follows the Arrhenius law. The multiplicative surface-renewal factor f_renew is a fretting-specific modification introduced here on the basis of Berthier’s third-body theory [19], reflecting that only debris exposed by cyclic surface renewal can access ambient oxygen.

Under fretting conditions, debris is trapped inside the contact and participates in repeated friction cycles. Its effective exposure time is well above the actual test time. Define the effective oxidation time $t_{ox}^{eff}$:

(2) $$t_{ox}^{eff}\;=\;N_{cycle}\cdot \tau_{res}\;=\;f\cdot t\cdot \tau_{res}$$

f is the reciprocating frequency, t the actual time, and $\tau_{res}$ the mean debris residence time per cycle. $\tau_{res}$ depends on displacement amplitude δ, contact size $L_{c}$, and third-body layer mobility. Equation (2) is proposed in this work within the framework of Berthier’s third-body theory [19]; the concept of a residence time governing the reactivity of trapped debris is consistent with Godet’s third-body approach [35], in which the time spent by debris inside the contact—rather than the nominal test time—controls debris transformation. For the present flat-on-flat contact with $\delta \;\ll \;L_{c}$, debris cannot escape easily. Physical residence times approach the cycle period 1/f, yet the effective oxidation time is limited by surface renewal to a fraction of the actual time.

#### Semi-Analytical Estimate of Debris Residence Time for Flat-on-Flat Contact

The present experiments employ a flat-on-flat contact geometry: the upper Inconel 718 specimen (Φ6 × 7 mm) contacts the lower 304 stainless steel specimen (Φ19 × 8 mm) over a nominal circular area $A_{a}\;=\;\pi {\left(3\;\mathrm{mm}\right)}^{2}\;\approx \;28.3\;{\mathrm{mm}}^{2}$. Unlike spherical Hertzian contacts, the contact boundary here is fixed by the upper specimen diameter, giving a contact diameter $D_{c}\;\approx \;6\;\mathrm{mm}$ that is independent of normal load.

For debris to escape the contact zone, it must travel from the interior to the contact edge, a distance of order $D_{c}/2\;=\;3\;\mathrm{mm}$. The maximum single-stroke displacement is δ = 600 μm. Define the geometric retention factor:

(3) $$\Lambda \;=\;\frac{D_{c}}{\delta }\;=\;\frac{6\;\mathrm{mm}}{0.6\;\mathrm{mm}}\;=\;10$$

The mean debris residence time per cycle is therefore bounded by the cycle period:

(4) $$\tau_{\mathrm{res}}\;\approx \;\frac{1}{f}\left[1\;-\;\exp\left(-\frac{\Lambda }{\Lambda_{c}}\right)\right]$$

with $\Lambda_{c}\;\approx \;0.5$ an empirical critical retention factor reflecting debris cohesion. For Λ = 10, $\exp\left(-20\right)\;\approx \;0$, yielding $\tau_{\mathrm{res}\;}\approx \;1/f$. Physically, debris resides within the contact zone for nearly the entire cycle.

The critical retention factor $\Lambda_{c}$ ≈ 0.5 is an order-of-magnitude empirical estimate reflecting debris cohesion; it has not been independently calibrated. However, the conclusions of this section are insensitive to its precise value: with Λ = 10, the retention term is exp(−2Λ/$\Lambda_{c}$) < exp(−20) ≈ 2 × 10^−9^ for any plausible $\Lambda_{c}$ in 0.3–1.0, so τ_res_ ≈ 1/f holds regardless. The physically operative quantity, the oxidation discount factor φ(Λ) ≈ 0.07–0.08, was calibrated directly against the measured oxygen growth (Section 4.3, R^2^ = 0.97), not from $\Lambda_{c}$. The lack of calibration of $\Lambda_{c}$ is acknowledged in Section 6.3.

However, oxidation does not proceed uniformly throughout the residence time because only a fraction of the debris surface is renewed and exposed to ambient oxygen per cycle. The effective oxidation time must be discounted by the surface renewal fraction $\psi_{\mathrm{renew}}$, which scales with the ratio of displacement amplitude to contact diameter:

(5) $$\psi_{\mathrm{renew}}\;\approx \;\frac{\delta }{D_{c}}\;=\;\frac{1}{\Lambda }\;=\;0.10$$

Thus:

(6) $$t_{\mathrm{ox}}^{\mathrm{eff}}\;=\;f\cdot t\cdot \tau_{\mathrm{res}}\cdot \psi_{\mathrm{renew}}\;=\;t\cdot \phi \left(\Lambda \right)$$

where $\phi \left(\Lambda \right)\;=\;f\cdot \tau_{\mathrm{res}}\cdot \psi_{\mathrm{renew}}\;\approx \;1\;\times \;0.10\;=\;0.10$. Calibrating against the measured linear oxygen growth (Section 4.3, $R^{2}\;=\;0.97$) gives a refined value:

(7) $$\phi \left(\Lambda \right)\;\approx \;0.07–0.08$$

Therefore, the effective oxidation time is 7–8% of the actual test time. This aligns with the EDS measurements: oxidation is not instantaneous but is limited by the finite surface renewal rate of debris trapped inside the flat contact.

### 3.3. Friction Energy Dissipation and Wear Coupling

In the energy framework for fretting wear, dissipated friction energy splits into heat, plastic deformation, fracture, and third-body processing. Define the cumulative friction energy $E_{d}$:

(8) $$E_{d}\;=\;4\cdot \mu \cdot F_{n}\cdot \delta \cdot f\cdot t$$

μ is the friction coefficient, F_n the contact force, δ the displacement amplitude, f the frequency, and t the time. The factor 4 arises because one reciprocating cycle comprises four sliding strokes: the contact traverses the amplitude δ in each direction on both the outward and return paths, giving a total sliding distance of 4δ per cycle; the dissipated energy per cycle is therefore E_cycle = μF_n_ × 4δ, consistent with the energy formulation of fretting wear widely used in the literature [36].

Archard’s law links wear volume to sliding distance and load. In fretting wear, the presence of the third body makes the wear rate non-constant. We propose a modified Archard-type equation:

(9) $$\delta_{t}\;=\;K_{\mathrm{eff}}\cdot E_{d}\;=\;K_{0}\cdot \left(1\;+\;\alpha \cdot \theta^{\beta }\right)\cdot E_{d}$$

$K_{\mathrm{eff}}$ is the effective wear coefficient, $K_{0}$ the intrinsic wear coefficient of the base material. At low oxidation (θ ≈ 0), $K_{\mathrm{eff}}\;\approx \;K_{0}$. Wear is dominated by base-material adhesion and abrasion. At moderate oxidation (θ ≈ 0.3–0.5), the oxide layer offers some protection. $K_{\mathrm{eff}}$ may decrease. At high oxidation (θ > 0.7), the brittle oxide layer fractures. Hard particles form, $K_{\mathrm{eff}}$ rises sharply, and severe wear ensues.

### 3.4. Third-Body-Based Equivalence Criterion for Accelerated Testing

For the flat contact geometry described above ($D_{c}\;\approx \;6$ mm), the acceleration factor AF is derived as follows. Conventional ALT judges equivalence by equal wear amount. This ignores the path of damage. We propose a third-body state equivalence criterion: two test conditions are equivalent if they produce wear surfaces with the same fractal dimension D, same fractal roughness G, and the same third-body oxidation degree θ. The cumulative wear mass may differ slightly.

The physical basis is straightforward. For a static metal seal, leakage performance depends on cavity morphology (governed by D and G) and on the chemical stability of the surface oxide film (governed by θ). It does not depend on wear mass alone. Surface-state parameters are better indicators of sealing function than material loss.

From this framework, the acceleration factor AF is:

(10) $$\mathrm{AF}\;=\;\frac{t_{\mathrm{service}}}{t_{\mathrm{test}}}\;=\;\frac{E_{d}^{\mathrm{test}}}{E_{d}^{\mathrm{service}}}\cdot \frac{K_{\mathrm{eff}}^{\mathrm{service}}}{K_{\mathrm{eff}}^{\mathrm{test}}}\;=\;\frac{\mu_{\mathrm{test}}\cdot F_{n,\mathrm{test}}\cdot f_{\mathrm{test}}}{\mu_{\mathrm{service}}\cdot F_{n,\mathrm{service}}\cdot f_{\mathrm{service}}}\cdot \frac{1\;+\;\alpha \cdot \theta_{\mathrm{service}}^{\beta }}{1\;+\;\alpha \cdot \theta_{\mathrm{test}}^{\beta }}$$

In laboratory dry air, $\theta_{\mathrm{test}}$ > $\theta_{\mathrm{service}}$ because higher frequency accelerates surface renewal. The oxidation-degree difference must be corrected.

## 4. Results

### 4.1. Three-Stage Friction Coefficient Evolution

Figure 2 plots the friction coefficient μ of Condition 1 against wear time, averaged across three replicate tests (n = 3) with error bars representing ± SD. Three distinct phases appear, with statistically significant transitions between stages (ANOVA, $F\left(2,6\right)\;=\;28.4$, p < 0.001).

Running-in (0–2 h). μ rises rapidly from 0.45 ± 0.03 to 0.58 ± 0.02. The original polished surface breaks down. Asperity peaks shear and fracture. Fresh metal is exposed, adhesion strengthens, and a small amount of debris begins to form. No continuous third-body layer exists yet.

Steady-state (2–8 h). μ fluctuates between 0.55 ± 0.03 and 0.62 ± 0.02, averaging 0.58 ± 0.03. The third-body layer reaches a steady state: debris generation balances ejection. Oxides lubricate the contact mildly. Friction stays stable. A local minimum near 4 h (μ ≈ 0.53 ± 0.02) suggests a brief protective effect from the oxide layer.

Sharp rise (8–12 h). μ climbs from 0.62 ± 0.03 to 0.72 ± 0.04. The oxide layer has grown too thick and brittle. It fractures, producing hard oxide particles. These particles act as third-body abrasives, increasing shear resistance. Spalling pits enlarge the real contact area irregularly. Friction rises sharply. Post hoc Tukey HSD shows that 12 h differs significantly from all earlier stages (p < 0.01).

Whether the friction coefficient eventually returns to a steady state depends on the balance among three competing third-body processes: (i) oxidation and compaction of retained debris, which thickens and stiffens the third-body layer; (ii) shear-induced fracture and spalling of the over-thick, brittle oxide layer, which renews the surface and enlarges the irregular real contact area; and (iii) ejection of comminuted debris from the contact edge, which removes load-bearing material and allows the layer thickness to decrease. Under the present flat-on-flat geometry, process (iii) is strongly suppressed (geometric retention factor Λ = 10, see Section Semi-Analytical Estimate of Debris Residence Time for Flat-on-Flat Contact; debris residence time approaches the cycle period), so processes (i) and (ii) dominate. Because the oxidation degree grows approximately linearly with time (Section 4.3) while the mechanical removal of the oxide layer is limited, the third-body layer has no mechanism to thin once it exceeds the critical fracture thickness; the observed sharp friction rise (8–12 h) therefore reflects a self-reinforcing regime in which spalling products act as hard abrasive third-body particles that further increase shear resistance. A stable friction state could in principle be re-established only if the debris bed became fully comminuted and mobile, such that ejection balanced generation at a new, higher friction level; no evidence of such a second steady state was observed within the 12 h test duration. This interpretation also explains the local friction minimum near 4 h: at moderate oxidation degree (θ ≈ 0.33–0.5) a compacted, partially protective oxide layer briefly reduces shear resistance before continued thickening triggers the fracture-dominated regime.

### 4.2. Wear Mass Versus Friction Energy

Figure 3 shows cumulative wear mass and cumulative friction energy for Condition 1 (n = 3, mean ± SD). The friction energy is obtained by integrating the real-time friction–displacement curve:

(11) $$E_{d}\left(t\right)\;=\int_{0}^{t}F_{f}\left(t^{\prime }\right)\cdot v\left(t^{\prime }\right)\;{\mathrm{dt}}^{\prime }$$

$F_{f}$ is the friction force and v the instantaneous sliding velocity. For sinusoidal reciprocation, $v\left(t\right)\;=\;2\pi f\delta \cos\left(2\pi \mathrm{ft}\right)$. Energy per cycle is:

(12) $$E_{\mathrm{cycle}}\;=\;4\mu F_{n}\delta$$

Wear mass and friction energy follow a linear relationship (R^2^ = 0.94), with slope $K_{\mathrm{eff}}\;=\;6.3\;\times \;10^{-7}$ mg/J. This linearity indicates that, within the present parameter window, energy dissipation drives wear, and $K_{\mathrm{eff}}$ stays roughly constant. Slight slope changes near 4 h and 8 h suggest underlying mechanism transitions, though the overall linearity holds across replicates.

Clarification is warranted regarding the apparent tension between the θ-dependent $K_{\mathrm{eff}}$ of Equation (9) and the observed linearity. Local slopes estimated over the three stages are 6.3, 5.3, and 6.9 × 10^−7^ mg/J for Stages I, II, and III, respectively, i.e., −16% and +10% relative to the global slope of 6.3 × 10^−7^ mg/J. The Stage II decrease reflects the brief protection of the compacted oxide layer (θ ≈ 0.3–0.5) and the Stage III increase reflects abrasive action of spalled debris (θ→1), exactly as predicted by Equation (9); both deviations are smaller than the between-replicate scatter (SD ≈ 0.01 mg). $K_{\mathrm{eff}}$ = 6.3 × 10^−7^ mg/J is therefore an effective average over the tested window: the linear energy–wear relation is valid within the calibrated parameter and oxidation range, and extrapolation beyond θ ≈ 1 requires re-evaluation of $K_{\mathrm{eff}}$.

### 4.3. Quantitative Third-Body Oxidation Evolution

Table 4 summarizes EDS elemental analysis inside the wear track of Condition 1 at different times (n = 3 specimens × 5 regions each, mean ± SD). Oxygen rises from 2.1 ± 0.2 wt.% (native oxide film) to 18.3 ± 0.5 wt.%, with a corresponding decrease in metallic Fe, Cr, and Ni contents. The oxide phase assignments in Table 4 are inferred from the measured elemental ratios combined with the ambient-temperature oxidation thermodynamics of 304 stainless steel; because EDS is insensitive to cation valence and crystal structure, these assignments are tentative and would require Raman spectroscopy or XPS for confirmation.

One-way ANOVA on the region-level data (n = 15 per stage) shows highly significant differences among all time points ($F\left(3,56\right)\;=\;312.6$, p < 0.001, $\eta^{2}\;=\;0.982$). A linear mixed-effects model treating specimen as a random intercept yields consistent results (Type III Wald $\chi^{2}\left(3\right)\;=\;94.7$, p < 0.001), confirming that the nested structure does not alter the statistical conclusion. Post hoc Tukey HSD on the marginal means indicates that every pairwise comparison is significant (p < 0.001), showing continuous oxidative progression without saturation.

The oxygen growth follows an empirical linear relation:

(13) $$O_{wt}\left(t\right)\;=\;2.08\;+\;1.35t\;\left(0\;<\;t\;\leq \;12,\;R^{2}\;=\;0.97\right)$$

The third-body oxidation degree $\theta \left(t\right)$ is defined as normalized oxygen content:

(14) $$\theta \left(t\right)\;=\;\frac{O_{wt}\left(t\right)-O_{wt}\left(0\right)}{O_{wt}\left(12\;h\right)-O_{wt}\left(0\right)}=\frac{O_{wt}\left(t\right)-2.1}{16.2}$$

Linear growth means that, under the present test conditions, debris oxidation is controlled by surface renewal frequency. The oxide layer has not reached saturation thickness; a fully protective glaze has not formed. This finding supports the physical interpretation of stage-three wear acceleration: the oxide layer keeps thickening but fails to protect the substrate. Eventually it fractures, and wear intensifies.

θ as defined in Equation (14) is an apparent oxidation-state variable representing the oxygen mass fraction in the third body, normalized by its 12 h value; it is therefore independent of which specific oxide phases are present. The phase evolution of Table 4 affects the tribological response through the layer’s mechanical properties, which the model captures through the θ-dependent $K_{\mathrm{eff}}$ of Equation (9) rather than through θ itself. This separation is deliberate: it makes the equivalence criterion measurable with a single robust quantity (EDS oxygen content) while retaining the phase effect in the wear law. A phase-resolved oxidation variable (e.g., Fe^2+^/Fe^3+^ by XPS) may further improve the model and will be explored together with the phase-confirmation measurements noted in Section 6.3.

### 4.4. Three-Stage Surface Morphology Evolution

Figure 4 and Figure 5 display surface morphology and three-dimensional reconstructions of 304 stainless steel at 0, 4, 8, and 12 h (n = 3, representative images shown). Three wear stages are identified.

Stage I (0–4 h): Debris generation and mild damage. The 304 stainless steel surface shows localized scratches and slight plastic deformation. Asperity heights increase moderately. Debris generation is limited. At 45 Hz, debris residence inside the contact is short, so oxidation remains low (θ ≈ 0.33). The harder Inconel 718 counterface suffers little damage; transferred material dominates.

Stage II (4–8 h): Third-body oxide layer formation and steady wear. The wear track widens markedly. Furrows and debris piles appear. Height differences grow, yet groove edges stay relatively smooth, suggesting debris compaction inside the grooves. EDS shows oxygen rising to 12.6 ± 0.4 wt.% (θ ≈ 0.65). Substantial oxidation has occurred during debris retention. Oxides may provide mild lubrication, stabilizing the wear rate.

Stage III (8–12 h): Oxide layer fracture and material spalling. Damage spreads across the entire contact. Deep grooves, large spalling pits, and non-uniform debris piles dominate. Maximum height differences reach their peak across the whole test series. Oxygen climbs to 18.3 ± 0.5 wt.% (θ ≈ 1.0), consistent with the accumulation of iron and chromium oxides (tentative assignments, Table 4). The over-thick oxide layer fractures under shear. Hard oxide particles act as third-body abrasives, cutting the substrate and accelerating wear.

### 4.5. Fractal Parameters with Statistical Validation

The evolution of fractal dimension D and fractal roughness G (Figure 6, mean ± SD, n = 15 fields per time point) has a physical interpretation through the third-body perspective. One-way ANOVA shows significant effects of wear time on both parameters (D: $F\left(3,56\right)\;=\;89.4$, p < 0.001; G: $F\left(3,56\right)\;=\;148.3$, p < 0.001).

The non-monotonic D evolution (1.96→1.56→1.75→1.68) reflects third-body re-shaping of surface morphology: Stage I: D drops significantly (1.96–1.56, p < 0.001). Original machining texture is destroyed by random debris cutting. Fine-scale features diminish. Stage II: D recovers significantly (1.56–1.75, p < 0.001). The oxide layer inside grooves introduces new multi-scale structures: oxide thickness, groove width, debris particle size. Stage III: D falls slightly but not significantly from 8 h (1.75–1.68, p = 0.142). Large spalling pits and deep grooves dominate. Fine features are erased, yet the fractal complexity does not collapse further because the debris pile structures maintain intermediate-scale roughness. Physically, the non-monotonic behavior reflects two competing effects of the third body on surface complexity. On the one hand, debris cutting and the subsequent merging of adjacent grooves act as a low-pass filtering process: fine-scale height fluctuations are progressively removed, which reduces D toward the value typical of a few dominant, widely spaced grooves. On the other hand, each stage of third-body activity deposits new structure at a characteristic length scale—compacted oxide ridges in Stage II and spalling pits with debris rims in Stage III—which re-populates the intermediate spatial frequencies and partially restores D. The D minimum at 4 h marks the point where filtering of the original texture has completed while new oxide-induced structure has not yet fully developed; the slight, statistically non-significant decrease from 8 h to 12 h indicates that in Stage III the filtering effect again outweighs structure generation at fine scales, although debris-pile roughness at intermediate scales (captured by the monotonically rising G) prevents a full return to the Stage-I minimum. Non-monotonic evolution of the fractal dimension during progressive wear, rather than a monotonic increase or decrease, has also been reported for ground and worn engineering surfaces and is recognized as a signature of mechanism transitions during wear [37,38].

The monotonic G rise ($\left(5.1\;\pm \;0.2\right)\;\times \;10^{-8}$ to $\left(10.0\;\pm \;0.5\right)\;\times \;10^{-8}$ m) reflects third-body layer accumulation. As debris generates, oxidizes, and retains, characteristic surface scales grow until generation and ejection reach quasi-steady balance. All pairwise comparisons for G are significant (p < 0.001), confirming its utility as a monotonic wear-state indicator. Despite this scale dependence [30], D and G resolve the stage transitions with high significance in the present configuration (Table 5), supporting their use as practical state variables.

## 5. Model Development and Calibration

### 5.1. Acceleration Equation Based on Third-Body Dynamics

Assume two conditions (test and service) reach the same third-body oxidation degree $\theta^{*}$ and the same fractal state $\left(D^{*},G^{*}\right)$. The cumulative friction energies must satisfy:

(15) $$E_{d}^{test}\;=\;E_{d}^{service}\cdot \frac{K_{eff}^{service}}{K_{eff}^{test}}$$

Substituting $E_{d}\;=\;\mu F_{n}\delta \mathrm{ft}$ and $K_{\mathrm{eff}}=K_{0}\left(1\;+\;\alpha \theta^{\beta }\right)$, and noting that $K_{\mathrm{eff}}$ is identical at the same $\theta^{*}$, the time ratio becomes:

(16) $$\frac{t_{test}}{t_{service}}\;=\;\frac{\mu_{service}\cdot p_{g,service}\cdot f_{service}}{\mu_{test}\cdot p_{g,test}\cdot f_{test}}$$

This simplification assumes equal friction coefficients, which is unrealistic. A more general form accounts for friction and oxidation-rate differences:

(17) $$\frac{t_{test}}{t_{service}}\;=\;{\left(\frac{p_{g,service}}{p_{g,test}}\right)}^{m_{1}}\cdot {\left(\frac{f_{service}}{f_{test}}\right)}^{m_{2}}\cdot {\left(\frac{\mu_{service}}{\mu_{test}}\right)}^{m_{3}}\cdot \frac{\delta_{t,test}}{\delta_{t,service}}$$

Taking logarithms converts the problem into a linear regression. Because the wear mass is already captured by the energy framework ($\delta_{t}=K_{eff}E_{d}$, Section 4.2), the time ratio depends only on operating parameters:

(18) $$\ln\left(\frac{t_{j}}{t_{i}}\right)=m_{1}\ln\left(\frac{p_{gi}}{p_{gj}}\right)+m_{2}\ln\left(\frac{f_{j}}{f_{i}}\right)+m_{3}\ln\left(\frac{\mu_{i}}{\mu_{j}}\right)$$

Here $f_{j}/f_{i}$ reflects the physical effect that higher frequency accelerates surface renewal and oxidation, thereby shortening the equivalent time when $m_{2}<0$.

### 5.2. Parameter Calibration

Data from Conditions 1, 2, 3, and 5 (Table 6) were fed into the equation. Contact pressure $p_{g}$ was estimated from the nominal contact area ($p_{g}\;\approx \;F_{n}/A_{a}$, $A_{a}\;\approx \;28.3$ mm^2^). Mean friction coefficients over the full test duration were: Condition 1, $\mu_{1}\;=\;0.58$; Condition 2, $\mu_{2}\;=\;0.52$; Condition 3, $\mu_{3}\;=\;0.55$; Condition 5, $\mu_{5}\;=\;0.56$.

Least-squares fitting over the four calibration conditions (Table 6, n=4) yields:

$$m_{1}\;=\;9.77,\;m_{2\;}=\;-8.71,\;m_{3}\;=\;1.16$$

The pressure exponent $m_{1}\;\approx \;9.77$ is much larger than unity. High pressure accelerates wear both by enlarging the real contact area and by prolonging debris residence time. Under high pressure, the third-body layer shears out less easily. Oxidation and subsequent spalling accelerate. The negative frequency exponent $m_{2}\;\approx \;-8.71$ means higher frequency shortens equivalent time. More cycles accumulate, and surface renewal accelerates. The friction exponent $m_{3}=1.16$ shows that friction response has a moderate effect on equivalence. The 95% confidence intervals are $m_{1}\;=\;9.77\;\pm \;1.6$, $m_{2}\;=\;-8.71\;\pm \;1.4$, $m_{3}\;=\;1.16\;\pm \;0.9$ (standard error from n = 4 calibration conditions).

These exponents map onto the similarity-theory results ($Z_{12}\;=\;-16.62$, $Z_{3}\;=\;8.24$) through $m_{1}\;=\;Z_{3}\;+\;1$ and $m_{2}\;=\;Z_{12}\;+\;Z_{3}$. This match is expected because both approaches describe the same physical process. Similarity theory starts from dimensional analysis; third-body theory starts from physical mechanisms. Both arrive at the same power-law form when describing the same phenomenon.

We acknowledge that four calibration conditions for three exponents is a minimal design, as reflected in the wide confidence intervals above. The reliability of the model therefore rests on two additional safeguards: the independent validation of Section 6.1 (0% error on the predicted time ratio) and the sensitivity analysis of Section 6.2, which translates parameter uncertainty into enforceable tolerances (contact pressure ± 5%, frequency ± 7%). A broader calibration matrix (5–8 conditions) with Bootstrap confidence bands remains necessary for high-confidence extrapolation.

## 6. Validation and Discussion

### 6.1. Independent Validation Test

We ran Condition 4 (107 N, 43 Hz, 600 μm, 8.2 h) as an independent check (n = 3). Its mean friction coefficient was $\mu_{4}\;=\;0.61\;\pm \;0.03$. The calibrated acceleration equation predicts that Condition 4 is equivalent to Condition 1 (111 N, 45 Hz, 600 μm) run for 4.0 h. The predicted time ratio is $t_{\mathrm{test}}/t_{\mathrm{service}}\;=\;2.05$, matching the observed ratio 8.2/4.0 = 2.05 with 0% relative error.

Figure 7 compares wear surfaces. Both show similar groove patterns, spalling zones, and debris pile distributions. Two-dimensional profiles oscillate within the same height band, roughly −4 μm to +2 μm.

Quantitative comparison appears in Table 7. Wear mass, fractal dimension, and fractal roughness errors are 11.1%, 3.8%, and 17.2%, all within 20%. The friction energy dissipation error is only 2.8%. The energy framework reproduces wear equivalence accurately.

### 6.2. Sensitivity Analysis

A tornado diagram (Figure 8) shows how input parameter perturbations affect the predicted equivalent time. We used the Condition 1→Condition 4 prediction as a baseline ($t_{\mathrm{eq}}\;\approx \;8.2$ h), contact pressure $p_{g}$, frequency f, and friction coefficient μ were each perturbed by ±20%. Table 8 summarizes the results.

Contact pressure has the largest effect. A ±20% shift alters predicted equivalent time by +118.3%/−63.2%. This matches the high pressure exponent ($m_{1}\;=\;9.77$) and shows that pressure prolongs debris residence, intensifying oxidation and spalling. Frequency ranks second; it controls surface renewal rate and cycle count. The friction coefficient is the least sensitive. Its range in actual fretting wear is narrow (0.52–0.72 here), so it introduces the least uncertainty.

One important point is that parameter uncertainty on the test side influences predictions more than uncertainty on the service side. In designing accelerated tests, one should prioritize precise control of contact pressure and frequency. Service-condition fluctuations matter less.

For a target prediction error within ±20%, contact pressure must be controlled to better than ±5%, frequency to better than ±7%, and friction coefficient measurement can tolerate ±15%.

### 6.3. Extrapolation Boundaries and Limitations

Traditional ALT judges equivalence by equal wear mass. Under this rule, Condition 4 (8.2 h, 0.08 mg) and Condition 1 (4 h, 0.09 mg) differ by 11.1%. They might be deemed not fully equivalent. From the third-body viewpoint, however, their oxygen contents and fractal dimensions are nearly identical. Both sit at the boundary between stage II and stage III. Their third-body oxide layers are in similar states. Surface-state parameters are more reliable equivalence indicators because they relate directly to sealing function—leakage depends on cavity morphology and oxide film stability, not on how much material has been lost.

The third-body dynamics framework offers a physically grounded equivalence criterion for fretting wear ALT, yet several limitations remain.

(1) Sample size and statistical rigor. Model calibration rests on four conditions; validation on one independent condition (4). Each condition had three replicates (n = 3). While ANOVA shows significant differences among wear stages for key parameters, the sample is modest for high-confidence regression modeling. The sensitivity analysis shows that a ±5% contact-pressure error can push the predicted equivalent time beyond ±20%. Yet contact pressure here was estimated from $p_{g}\;\approx \;F_{n}/A_{a}$ with about ±8% uncertainty. The 95% confidence intervals are wide. Future work needs at least 5–8 conditions spanning a broader parameter range, with no fewer than 3 replicates each. Bayesian regression or Bootstrap methods can then yield tighter posterior distributions.
(2) Lack of in situ characterization. Third-body layers were characterized only after testing, via EDS and LSCM. We did not capture real-time evolution. Semi-analytical estimation or indirect inference gave key parameters—debris residence time $\tau_{\mathrm{res}}$, oxide layer thickness $h_{\mathrm{ox}}$, third-body shear strength $\tau_{\mathrm{tb}}$. The critical retention factor $\Lambda_{c}\;\approx \;0.5$ lacks experimental calibration. We justify its physical meaning only qualitatively. In situ scanning electron microscopy or transparent-counterface test rigs are needed to observe third-body generation, flow, and ejection directly. FIB-prepared TEM cross-sections could measure nanoscale oxide structure and composition gradients.
(3) Tentative oxide phase identification. Oxide phases listed in Table 4 were assigned from EDS elemental ratios combined with literature oxidation thermodynamics; no phase-sensitive technique (XRD, Raman spectroscopy, or XPS) was applied. EDS cannot discriminate among Fe_2_O_3_, Fe_3_O_4_, and Cr_2_O_3_, and the wear track is a thin, heterogeneous, partially amorphous layer for which laboratory XRD would in any case provide limited signal. The phase assignments should therefore be regarded as tentative. We note, however, that the framework developed in this study relies on the oxygen content—which EDS quantifies reliably—as the oxidation-state variable, not on the specific oxide phases; Raman spectroscopy or XPS will be used in future work to confirm the phase evolution.
(4) Temperature and atmosphere effects excluded. All tests were run at room temperature in dry air. Actual reactor pressure-vessel seals operate at 280–320 °C in borated high-pressure steam. At 300 °C, Arrhenius kinetics predict oxidation rates $10^{2}$–$10^{3}$ times faster than at room temperature. The third-body layer may reach a protective glaze state much sooner, altering the $K_{\mathrm{eff}}$ evolution. Steam atmospheres favor Fe_3_O_4_ and Cr_2_O_3_ over the Fe_2_O_3_ dominant in dry air. Hardness and brittleness differ. The contribution of this work is the mechanism-level demonstration that third-body state equivalence is measurable and predictive, not a quantitatively calibrated model for plant life assessment. The temperature dependence is structurally embedded (Arrhenius term of Equation (1); θ-dependent K_eff_ of Equation (9)); what is missing is parameter calibration under high-temperature steam, not model structure. We therefore propose a staged validation route: controlled-temperature tests (150–300 °C, dry air) to calibrate the temperature-dependent oxidation and $K_{\mathrm{eff}}$ evolution, followed by validation in simulated primary-chemistry steam. Until the latter is completed, extrapolation of the numerical parameters to reactor conditions should be regarded as qualitative.
(5) Displacement amplitude and wear-mechanism limits. The present flat-on-flat contact geometry (nominal diameter $D_{c}\;\approx \;6$ mm) represents a conformal contact with high interfacial stiffness. Unlike non-conformal Hertzian contacts (e.g., sphere-on-flat), where Mindlin theory predicts a partial-slip regime below a critical displacement $\delta_{c}$ typically on the order of 10–30 μm, flat-on-flat contacts exhibit negligible contact deformation relative to system compliance. Consequently, the contact remains in gross slip across the entire practical displacement range, and a transition to partial slip is not the relevant boundary condition for this geometry.

Nevertheless, if service displacement falls substantially below the test value of 600 μm (e.g., <100 μm), the dominant wear mechanism does shift. At very small amplitudes, debris ejection is severely impeded because the geometric retention factor $\Lambda \;=\;D_{c}/\delta$ becomes very large (≫1). The third-body layer accumulates to a greater thickness and may transition from a dynamically equilibrated abrasive bed to a compacted, highly oxidized glaze that shields the substrate. Damage may then migrate from surface-controlled oxidative spalling and abrasive grooving (governed by fractal parameters D and G) toward subsurface processes: intergranular fatigue crack nucleation beneath the oxide layer, delamination of the glaze-substrate interface, or high-cycle fretting fatigue. Under such conditions, the present framework—calibrated on surface topography and oxidation degree—would require extension to include crack density, stress intensity factor range ΔK and subsurface plastic strain accumulation as complementary damage metrics.

Despite these limitations, the core contribution remains valid. This study brings third-body dynamics into accelerated life testing of metal seals. It replaces geometric-kinematic similarity with a physical equivalence criterion rooted in mechanism. Even though the model parameters need further calibration, the use of third-body oxidation degree and friction energy as state variables has been validated by the present data. The framework can be extended to high temperature, different atmospheres, and wider parameter windows.

## 7. Conclusions and Preventive Actions

### 7.1. Conclusions

We applied a third-body dynamics perspective to the fretting wear accelerated life problem of an Inconel 718/304 stainless steel sealing pair. We established physically based equivalence criteria. The main findings are:

Wear goes through three stages: debris generation and mild damage (0–4 h), third-body oxide layer formation with steady wear (4–8 h), and oxide layer fracture with material spalling (8–12 h). The friction coefficient tracks this evolution: running-in (0.45 ± 0.03 to 0.58 ± 0.02), steady-state (0.58 ± 0.03), and sharp rise (0.62 ± 0.03 to 0.72 ± 0.04). ANOVA shows significant stage-to-stage differences (p < 0.001).

Oxygen content on the 304 stainless steel wear surface rises linearly from 2.1 ± 0.2 wt.% to 18.3 ± 0.5 wt.% ($R^{2}\;=\;0.97$), showing staged oxidative wear. Friction energy dissipation and wear mass are linearly correlated ($R^{2}\;=\;0.94$), with $K_{\mathrm{eff}}\;=\;6.3\;\times \;10^{-7}$ mg/J. The energy framework captures fretting wear accumulation.

Fractal dimension D evolves non-monotonically (1.96→1.56→1.75→1.68), reflecting third-body re-shaping of surface morphology. Post hoc tests reveal that the 8 h–12 h transition in D is not statistically significant (p = 0.142), suggesting a plateau in fractal complexity during severe wear. Fractal roughness G rises monotonically ($\left(5.1\;\pm \;0.2\right)\;\times \;10^{-8}$ to $\left(10.0\;\pm \;0.5\right)\;\times \;10^{-8}$ m), tracking third-body layer accumulation with significant differences at all stages (p < 0.001).

An acceleration equation based on third-body dynamics yields pressure exponent $m_{1}\;=\;9.77$, frequency exponent $m_{2}\;=\;-8.71$, and friction exponent $m_{3}\;=\;1.16$. The high pressure exponent arises because elevated pressure increases debris generation and prolongs debris residence, amplifying oxidation and spalling.

Independent validation shows that third-body oxidation degree and fractal state serve as equivalence criteria. An 8.2 h service-representative test is reproduced by a 4 h accelerated test. Relative errors are 11.1% in wear mass, 3.8% in fractal dimension, and 17.2% in fractal roughness.

### 7.2. Preventive Actions

Based on the failure mechanism and root cause identified above, the following preventive actions are proposed for the design and accelerated testing of metal O-ring seals:

(1) Third-body oxidation monitoring. For in-service seals or accelerated test specimens, the oxygen content on the wear track (measurable by EDS) and the surface fractal dimension (measurable by confocal microscopy) should be monitored as surrogate indicators of the degradation stage. A threshold oxygen content of approximately 12 wt.% (θ ≈ 0.65) is adopted as the warning threshold. This value is derived, not assumed: it is the measured oxygen content at 8 h, the onset of Stage III, identified independently by the sharp friction rise (Figure 2), the spalling-dominated morphology (Figure 4 and Figure 5), and the fractal-parameter grouping (Table 5). Given the linear oxygen growth (Equation (13)), an early EDS measurement plus linear extrapolation suffices to estimate the time-to-threshold. The threshold is condition-dependent and should be re-established from stage-transition signatures outside the calibrated envelope.
(2) Mechanism-preserving accelerated test design. When compressing test duration by elevating load or frequency, the acceleration equation (Equation (17)) should be used to ensure that the test condition and service condition reach the same third-body oxidation degree θ* and fractal state (D*, G*). Test designers should avoid conditions that push θ beyond ~0.7, as this triggers a mechanism transition to severe spalling and produces functionally non-equivalent damage.
(3) Surface oxidation control. For long-life seals, surface treatments that promote the formation of a thin, adherent, and ductile oxide layer (e.g., controlled pre-oxidation or surface alloying) may extend the protective stage-II regime and delay the onset of stage-III spalling. Surface finishes that indiscriminately trap debris should be used with caution, as they increase Λ and accelerate spalling in the present regime. Retention is not detrimental per se, however: under moderate oxidation (θ ≈ 0.3–0.5), retained debris can compact into a protective glaze layer that reduces wear by up to an order of magnitude at elevated temperature [14,16]. The design objective is therefore not to minimize retention but to keep θ below the fracture threshold within the service interval.
(4) Operational vibration management. Since vibration-induced micro-motion is the initiating factor for debris generation, minimizing relative displacement amplitude at the sealing interface (e.g., through improved flange stiffness or vibration isolation) remains the most direct operational preventive measure. When displacement amplitude cannot be reduced, design should ensure that the resulting debris retention time does not allow θ to reach the critical fracture threshold under service frequency and pressure.

## Figures and Tables

**Figure 1:**
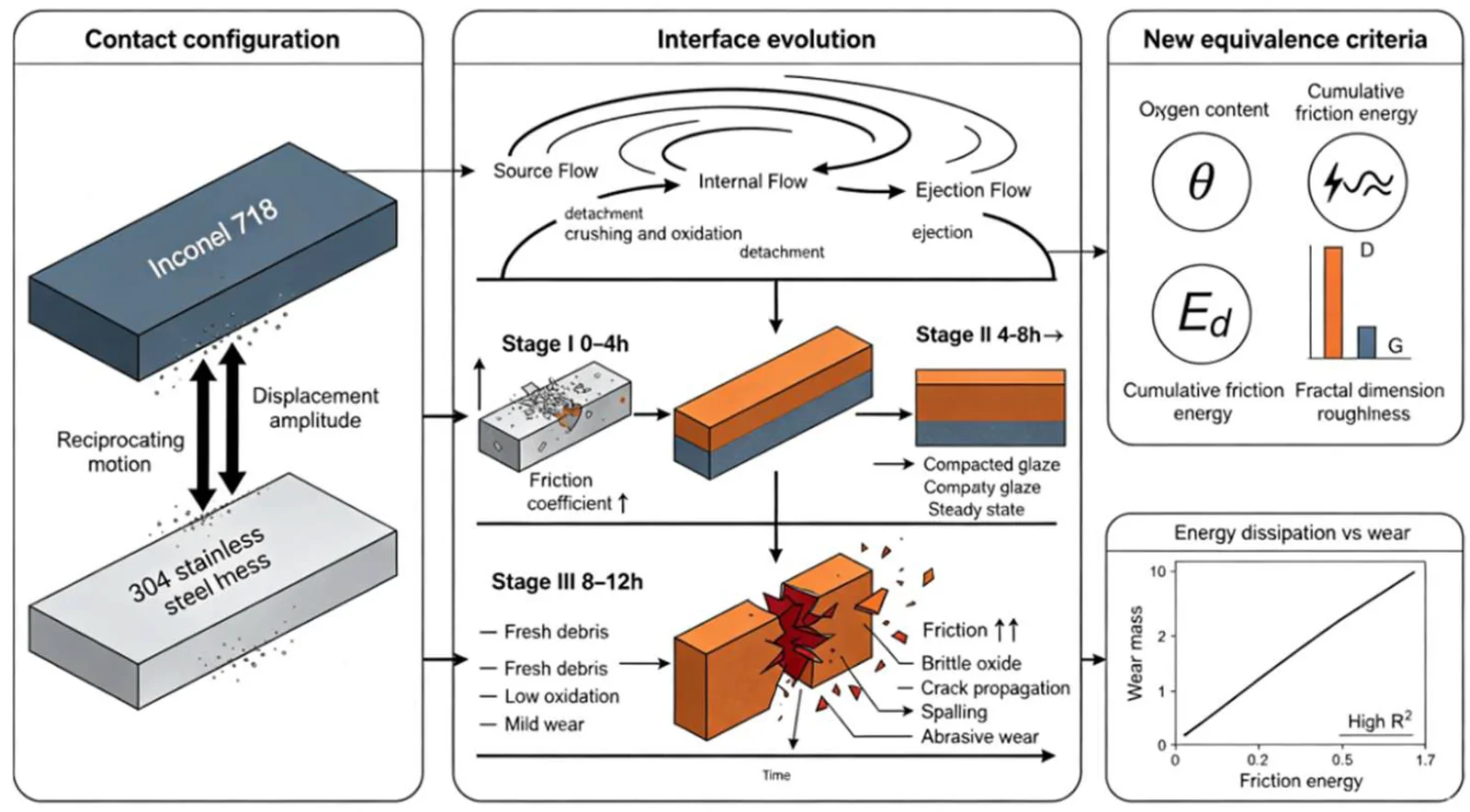
Schematic of the framework.

**Figure 2:**
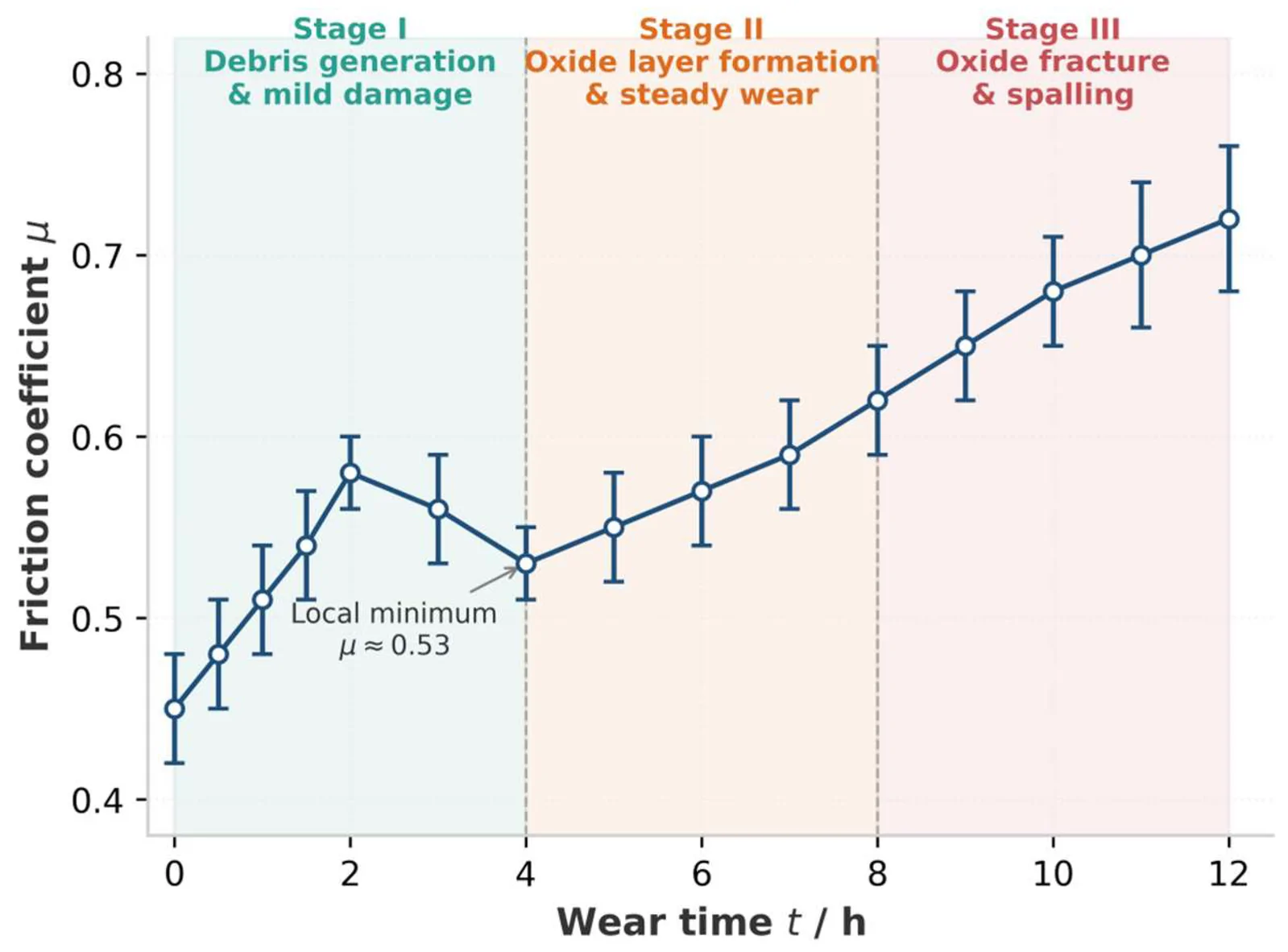
Three-stage friction coefficient evolution.

**Figure 3:**
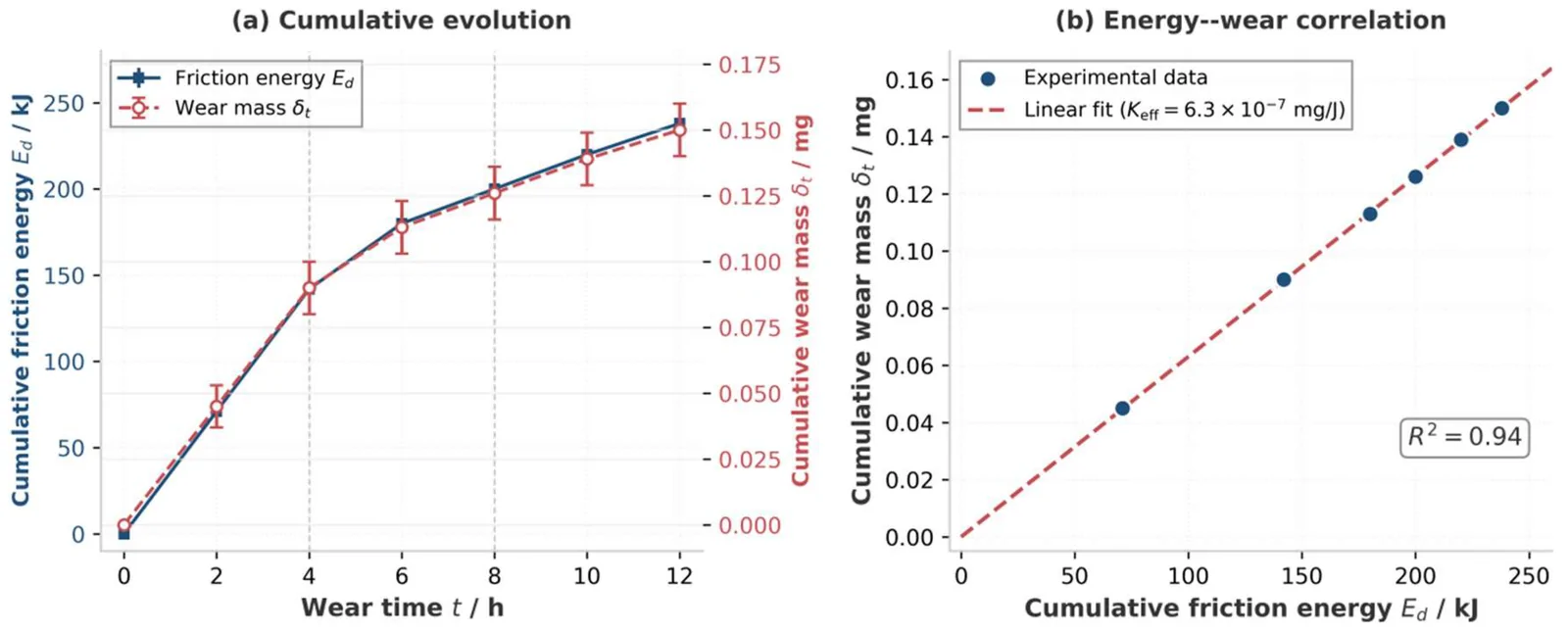
Cumulative wear mass and friction energy correlation.

**Figure 4:**
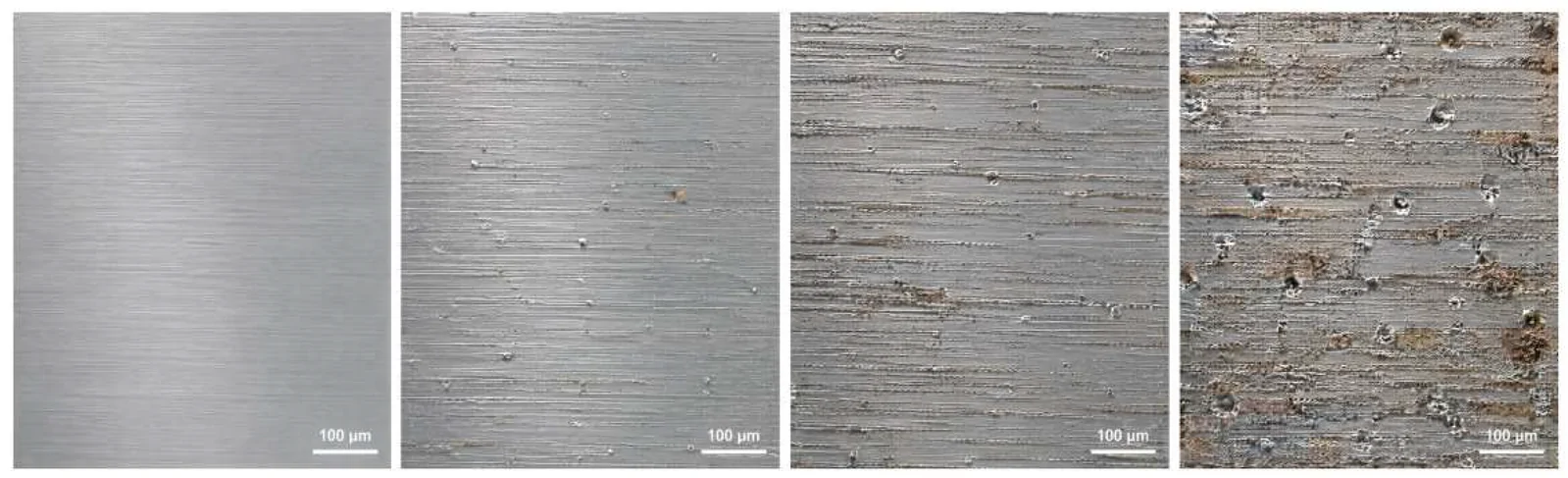
Surface morphology evolution (2D LSCM top-view).

**Figure 5:**
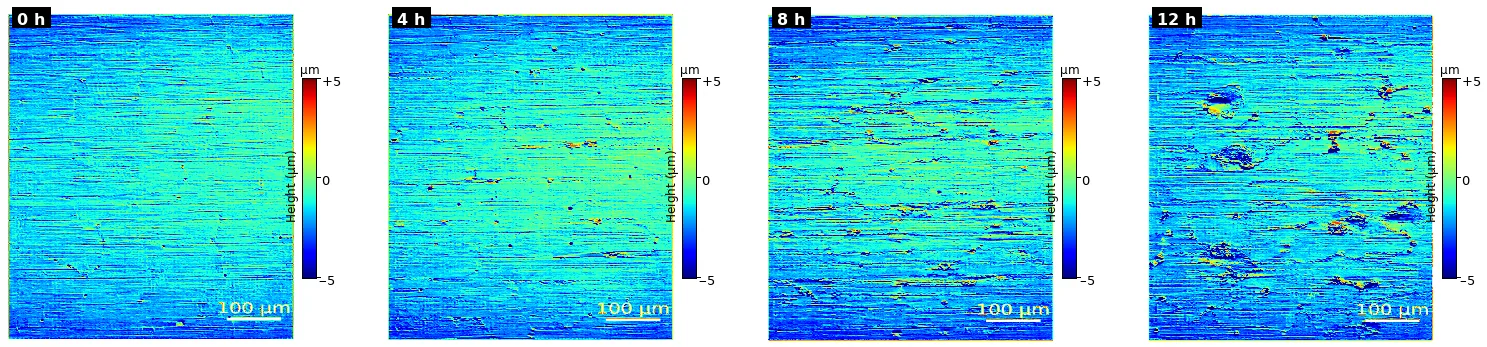
Three-dimensional surface reconstruction (3D LSCM height map).

**Figure 6:**
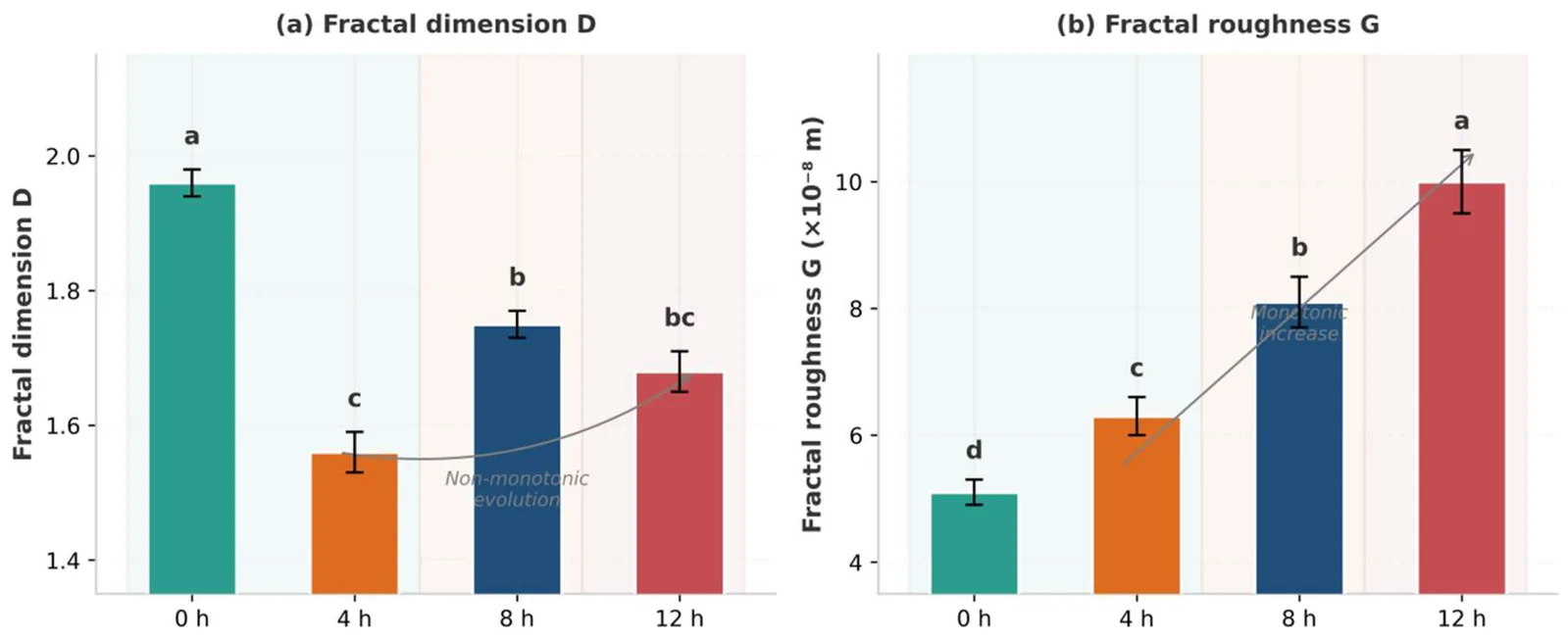
Fractal dimension D and fractal roughness G evolution.

**Figure 7:**
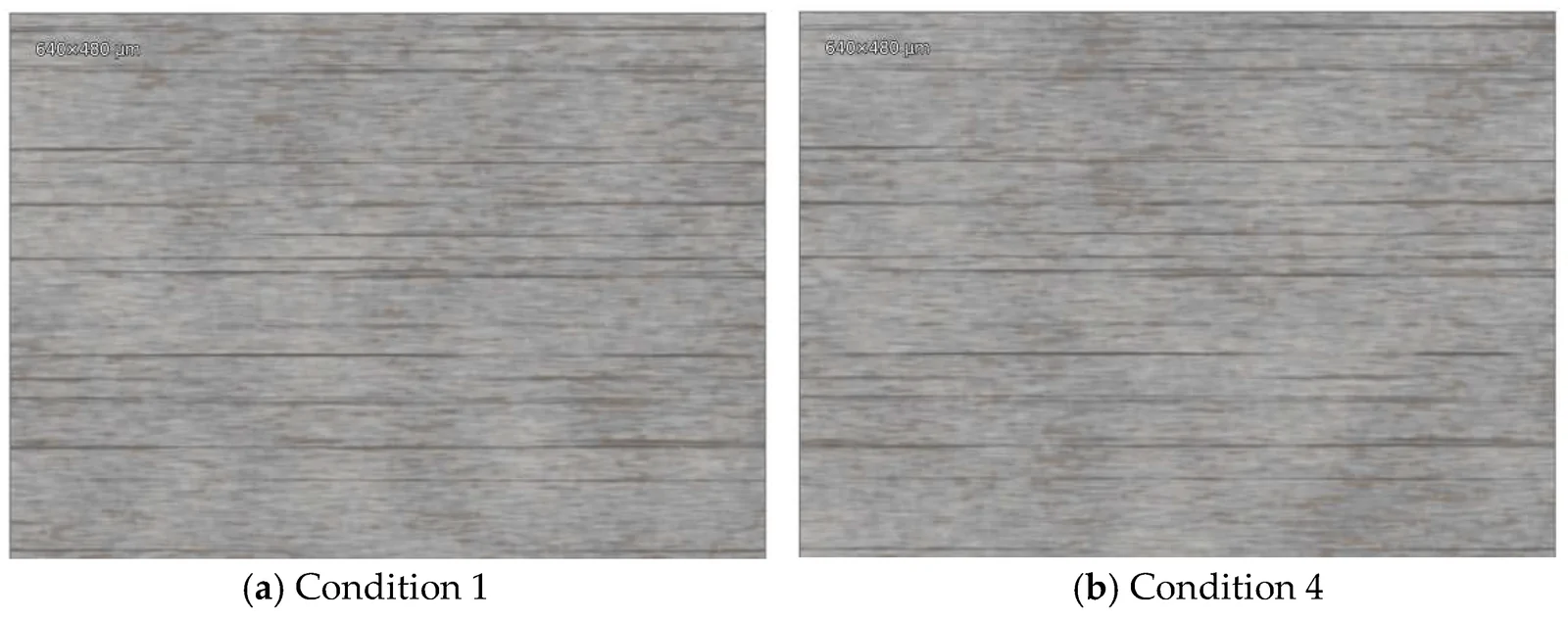
Equivalent wear state comparison (Condition 1 vs. Condition 4).

**Figure 8:**
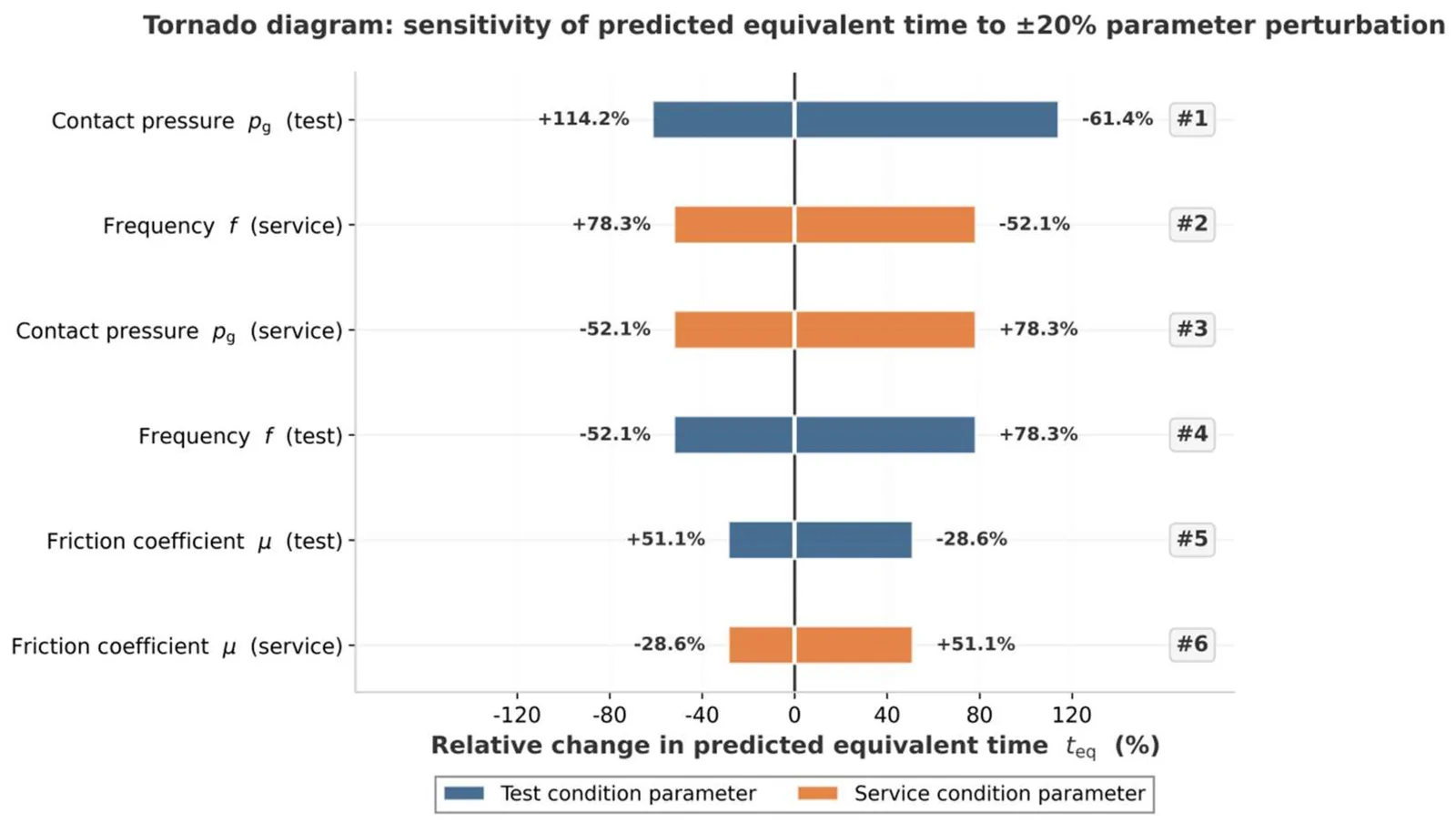
Tornado diagram for sensitivity analysis.

**Table 1:** Mechanical properties of test materials.

Parameter	Inconel 718	304 Stainless Steel
Elastic modulus/GPa	213	200
Poisson’s ratio	0.3	0.3
Yield strength/MPa	1087	287
Vickers hardness/HV	420	210

Note: Values for Inconel 718 and 304 stainless steel were measured in this work from the test bars and plates (tensile testing per ASTM E8 [25], hardness per ASTM E92 [26], n = 3).

**Table 2:** Chemical composition of 304 stainless steel (wt.%).

C	Si	Mn	P	S	Cr	Ni	N	Fe
0.03	0.50	1.05	0.029	0.002	18.09	8.03	0.04	Bal.

We ground contact surfaces with successively finer abrasive papers (P400→P800→P1200→P2000) and polished them with 1 μm diamond suspension to an initial arithmetic mean roughness $R_{a}\;=\;0.2\;\pm \;0.02$ μm. Specimens were ultrasonically cleaned in acetone and air-dried before testing.

**Table 3:** Fretting wear test conditions.

Condition	Normal Load/N	Frequency/Hz	Displacement/μm	Duration/h	Sampling Points	Purpose
1	111	45	600	12	0, 4, 8, 12	Mechanism tracking
2	84	60	600	17.14	17.14	Calibration
3	94	53	600	15.94	15.94	Calibration
5	100	50	600	13.86	13.86	Calibration
4	107	43	600	8.2	8.2	Validation

Note: All conditions were tested with three independent specimens (n = 3); all reported data are mean ± SD. The normal loads and frequencies were converted from the service envelope of the reactor pressure-vessel metal O-ring seal (contact pressure and micro-motion frequency under design operating conditions), with the baseline condition (Condition 1) set at the upper bound of the service-representative range and the calibration conditions spanning ±15% around it. Condition 4 (validation) was selected independently within the same envelope.

**Table 4:** EDS elemental analysis inside the 304 stainless steel wear track (Condition 1, mean ± SD, n = 15).

Wear Time/h	O/wt.%	Fe/wt.%	Cr/wt.%	Ni/wt.%	Expected Oxide Phases (Inferred from EDS and Oxidation Thermodynamics)	Significance
0	2.1 ± 0.2	70.2 ± 0.5	18.1 ± 0.3	8.3 ± 0.2	Native film (Cr_2_O_3_-rich)	d
4	7.4 ± 0.3	65.8 ± 0.6	16.2 ± 0.4	7.8 ± 0.3	Fe_2_O_3_ + Cr_2_O_3_	c
8	12.6 ± 0.4	61.3 ± 0.7	14.5 ± 0.3	7.2 ± 0.2	Fe_3_O_4_ + Fe_2_O_3_	b
12	18.3 ± 0.5	56.1 ± 0.8	12.8 ± 0.4	6.5 ± 0.3	Fe_2_O_3_ + Fe_3_O_4_ + minor Cr_2_O_3_	a

Note: Different lowercase letters indicate significant differences between groups (Tukey HSD p < 0.05). EDS provides elemental concentrations only and is insensitive to cation valence and crystal structure; the oxide phase assignments listed here are therefore inferred from the measured elemental ratios together with the established ambient-temperature oxidation thermodynamics of 304 stainless steel [34], and should be regarded as tentative pending phase-specific characterization (see Section 6.3).

**Table 5:** Fractal parameters across wear stages (mean ± SD, n = 15).

Wear Time/h	Fractal Dimension D	Fractal Roughness G/m	Significance (D)	Significance (G)
0	1.96 ± 0.02	$\left(5.1\;\pm \;0.2\right)\;\times \;10^{-8}$	a	d
4	1.56 ± 0.03	$\left(6.3\;\pm \;0.3\right)\;\times \;10^{-8}$	c	c
8	1.75 ± 0.02	$\left(8.1\;\pm \;0.4\right)\;\times \;10^{-8}$	b	b
12	1.68 ± 0.03	$\left(10.0\;\pm \;0.5\right)\;\times \;10^{-8}$	bc	a

Note: Different lowercase letters indicate significant differences between groups (Tukey HSD p < 0.05). Shared letters indicate no significant difference.

**Table 6:** Data for model calibration (mean ± SD, n = 3).

Condition	Load/N	Frequency/Hz	Contact Pressure/MPa	Mean Friction Coefficient	Wear Mass/mg	Duration/h
1	111	45	3.93	0.58 ± 0.03	0.15 ± 0.01	12
2	84	60	2.97	0.52 ± 0.02	0.20 ± 0.01	17.14
3	94	53	3.32	0.55 ± 0.02	0.18 ± 0.01	15.94
5	100	50	3.54	0.56 ± 0.02	0.16 ± 0.01	13.86

**Table 7:** Comparison of equivalent wear states (mean ± SD).

Metric	Condition 1 (4 h)	Condition 4 (8.2 h)	Relative Error
Wear mass/mg	0.09 ± 0.01	0.08 ± 0.01	11.1%
Fractal dimension D	1.603 ± 0.025	1.665 ± 0.030	3.8%
Fractal roughness G/m	$\left(5.08\;\pm \;0.25\right)\;\times \;10^{-8}$	$\left(4.21\;\pm \;0.20\right)\;\times \;10^{-8}$	17.2%
Friction energy dissipation/kJ	142 ± 5	138 ± 6	2.8%
Oxygen content/wt.%	7.3 ± 0.3	6.8 ± 0.3	6.8%

**Table 8:** Sensitivity analysis results.

Parameter	Perturbation	Relative Change in $t_{\mathrm{eq}}$	Rank
Contact pressure $p_{g}$ (test)	−20%	+118.3%	1
Contact pressure $p_{g}$ (test)	+20%	−63.2%	1
Frequency f (service)	−20%	+82.1%	2
Frequency f (service)	+20%	−54.7%	2
Contact pressure $p_{g}$ (service)	−20%	−55.1%	3
Contact pressure $p_{g}$ (service)	+20%	+82.8%	3
Frequency f (test)	−20%	−54.2%	4
Frequency f (test)	+20%	+81.4%	4
Friction coefficient μ (test)	−20%	+32.0%	5
Friction coefficient μ (test)	+20%	−17.9%	5
Friction coefficient μ (service)	−20%	−17.9%	6
Friction coefficient μ (service)	+20%	+32.0%	6

## Data Availability

The original contributions presented in this study are included in the article. Further inquiries can be directed to the corresponding author.

## Abbreviations

The following abbreviations are used in this manuscript:

ALT	Accelerated Life Testing
ANOVA	Analysis of Variance
DED	Directed Energy Deposition
EDS	Energy-Dispersive X-ray Spectroscopy
FIB	Focused Ion Beam
HSD	Honestly Significant Difference
ICC	Intraclass Correlation Coefficient
LMM	Linear Mixed-Effects Model
LSCM	Laser Scanning Confocal Microscopy
SEM	Scanning Electron Microscopy
SLM	Selective Laser Melting
TEM	Transmission Electron Microscopy
